# Dragon TIS Spotter: an Arabidopsis-derived predictor of translation initiation sites in plants

**DOI:** 10.1093/bioinformatics/bts638

**Published:** 2012-10-30

**Authors:** Arturo Magana-Mora, Haitham Ashoor, Boris R. Jankovic, Allan Kamau, Karim Awara, Rajesh Chowdhary, John A.C. Archer, Vladimir B. Bajic

**Affiliations:** ^1^King Abdullah University of Science and Technology (KAUST), Computational Bioscience Research Center, Thuwal 23955-6900, Saudi Arabia and ^2^Biomedical formatics Research Center, MCRF, Marshfield Clinic, Marshfield, WI 54449, USA

## Abstract

**Summary:** In higher eukaryotes, the identification of translation initiation sites (TISs) has been focused on finding these signals in cDNA or mRNA sequences. Using *Arabidopsis thaliana* (*A.t.*) information, we developed a prediction tool for signals within genomic sequences of plants that correspond to TISs. Our tool requires only genome sequence, not expressed sequences. Its sensitivity/specificity is for *A.t.* (90.75%/92.2%), for *Vitis vinifera* (66.8%/94.4%) and for *Populus trichocarpa* (81.6%/94.4%), which suggests that our tool can be used in annotation of different plant genomes. We provide a list of features used in our model. Further study of these features may improve our understanding of mechanisms of the translation initiation.

**Availability and implementation:** Our tool is implemented as an artificial neural network. It is available as a web-based tool and, together with the source code, the list of features, and data used for model development, is accessible at http://cbrc.kaust.edu.sa/dts.

**Contact:** vladimir.bajic@kaust.edu.sa

**Supplementary information**: Supplementary data are available at *Bioinformatics* online.

## 1 INTRODUCTION

One of the objectives of bioinformatics is to identify important biological signals in genomic sequences. The translation initiation site (TIS) is one such signal in mRNA that denotes the start codon at which translation initiates. Accurate recognition of TIS signals can help in discovery of protein-coding genes and improve annotation of gene loci (Do and Choi, 2006; Preiss and Hentze, 2003). In genomic DNA, signals that correspond to TISs consist of the ATG triplet of nucleotides, except for the rare cases of ACG or CTG triplets (Hann, 1994; Kozak, 1989). In this study, we focus solely on the recognition of ATG motifs within DNA that correspond to genuine TIS signals. We will refer to the ATG triplets as TIS motifs. Our study addresses prediction of TIS motifs in plant species. Recognizing DNA motifs in genomic sequences that correspond to genuine TIS signals is much more complex than recognizing them in mRNA or cDNA sequences, which was the main focus so far. Pertea and Salzberg achieved accuracy of 84% on both *Arabidopsis thaliana* (*A.t.*) and human genomic sequences (Pertea and Salzberg, 2002). Sparks and Brendel developed MetWAMer tools, achieving an accuracy of 85% on *A.t.* open reading frame sequences (Sparks and Brendel, 2008). In this study, using *A.t.* information, we developed a model for predicting TIS motifs within plant genomic DNA sequences, and we generated a number of features to characterize the genomic surroundings of these motifs. Some of these features have already been used for related tasks (Li and Leong, 2005; Liu and Wong, 2003), but we introduced a number of new features not previously used for TIS predictions. Out of all the features initially considered, we selected 47 as the best set of features for the TIS motif recognition task. Our feature selection is based on a wrapper method that uses a genetic algorithm (GA) and an Artificial Neural Network (ANN). There are other studies that deal with the generation and selection of features for the TIS recognition, for example (Zeng *et al.*, 2002). To the best of our knowledge, our TIS predictor is the only publicly available one for plants. The sensitivity/specificity of our model for *A.t.* is 90.75%/92.2% and is the highest compared with those reported in the literature. The accuracy tests on chromosomes of other plant genomes show sensitivity/specificity for *Vitis vinifera* of 66.8%/94.4%, and *Populus trichocarpa* of 81.6% / 94.4%. The web-based tool that implements our algorithm and our datasets are freely accessible at http://cbrc.kaust.edu.sa/dts.

## 2 METHODS

### 2.1 Datasets

TIS data for *A.t.* was extracted from *A.t.* genome and the corresponding annotations file obtained from the TAIR database, version 10 (http://www.arabidopsis.org). We extracted a total of 27 388 genuine TIS samples for positive dataset that correspond to database entries annotated as ‘protein coding gene’. The same number of false TIS samples was generated from *A.t.* chromosomes 1 to 5, ensuring that any such sequence is not present in the TIS-positive set. Positive and negative TIS sequences are 300 bp in length with the TIS covering positions 150–152 counted in 5′–3′ direction. The number of negative samples taken from each of the chromosomes was proportional to the chromosome size. Even though negative cases are far more prevalent in the genome, we used equal-sized positive and negative datasets for training because we believe that these data sets contain sufficiently rich distinguishing features to separate genuine TIS motifs from the false ones.

### 2.2 Feature generation

For TIS prediction, many useful features in sequences surrounding ATG signals are reported. Prominent amongst these can be found, for example, in (Li et al., 2004; Liu and Wong, 2003; Liu *et al.*, 2004; Ma *et al.*, 2006; Saeys *et al.*, 2007; Tzanis and Vlahavas, 2006). Many of the reported features are local in the sense that they primarily characterize properties of the sequences immediately surrounding a candidate TIS. We extended this set of features with some that are affected by nucleotides up to 150 bp from the ATG motif site. Since selection of the optimal combination of candidate features is a combinatorial problem, we first reduce the size of the search space by defining a predetermined subset of features used in all feature-selection iterations. This fixed subset consists of features that we selected based on the previously reported results for which we believe to play a significant role in TIS recognition. We expanded the considered features with a number of new ones. The feature selection method enlarged the fixed feature set. The core step in our feature selection process is the application of genetic algorithm (GA) in search of an optimal features combination. Briefly, the process stipulates that all candidate features are numbered and assigned a value of 0 (not selected as a member of a feature set) or 1 (selected). In this way we form a ‘chromosome’ in the GA terms. We use a single point crossover together with mutation where each bit in a chromosome is subjected to 15% chance of having its value altered. Finally, we define evaluation function as the accuracy of model based on a 3-fold cross-validation on the training data. Description of major features and more details on feature selection, training and testing, are given in Supplementary Material 1. A full list of the used features is given in Supplementary Material 2.

### 2.3 Main classifier

Our prediction model is an ANN-based classifier. ANNs were used before for TIS prediction (Pedersen and Nielsen, 1997; Rajapakse and Ho, 2005; Tikole and Sankararamakrishnan, 2008). We used a 31-node single hidden-layer ANNs and the backpropagation algorithm for weights optimization. After selecting features using GA, we train the ANN. Available data, 27 388 positive (real TISs) and 27 388 negative (false TISs) samples, are split into the training and testing sets. From each of these two sets, 65% (18 802) are reserved for model training and the remaining 35% (9586) for testing. The training data (18 802 positive and 18 802 negative samples) were further divided into three parts. The first one, containing 5000 positive and 5000 negative samples were exclusively used to generate feature values. The second set containing 10 882 positive and 10 882 negative samples is used for ANN training. To avoid overfitting, the early stopping with validation method (Prechelt, 1998) is used on the remaining 2920 positive and 2920 negative samples as a validation set.

## 3 RESULTS

As a representative measure of model performance, we used the model sensitivity defined as Se = TP/(TP + FN) and specificity Sp = TN/(TN + FP), where TP, TN, FP and FN are the numbers of true positive predictions, true negative predictions, false positive predictions and false negative predictions, respectively. When evaluated on the test data only, the performance of our TIS prediction model for *A.t.* resulted in Se = 90.75% and Sp = 90.77%. When we tested our model on the whole *A.t.* genome excluding the training data, we obtained Se = 90.75% and Sp = 92.2%. The tests of our TIS prediction in other plant genomes, with the unmodified Arabidopsis model, resulted on *Vitis vinifera* (entire chromosomes 1 and 2) in Se = 66.8% and Sp = 94.4%, and on *Populus trichocarpa* (entire chromosome 1) in Se = 81.6% and Sp = 94.4%. Details are in Supplementary Material 3.

## 4 CONCLUSION

We developed a web tool for the recognition of TIS motifs in plant genomic DNA sequences that is based on an ANN classifier. Model features are selected by a GA as a part of the model optimization process. The model demonstrates not only an improved prediction accuracy over the reported TIS predictors for *A.t*., but also performs well on two other plant species for which it was not specifically trained. We hope that our tool will find good use in studies and annotation of gene properties of plants and may provide a further insight into the mechanisms of translation initiation.

*Conflict of Interest*: none declared.

## Supplementary Material

Supplementary Data
