# Supplementary material for: Dragon TIS Spotter: an Arabidopsis-derived predictor of translation initiation sites in plants
Source: Bioinformatics. 2012 Oct 30;29(1):117–8. doi: 10.1093/bioinformatics/bts638 (PMC3530916; doi:10.1093/bioinformatics/bts638)
Supplement: Supplementary Data [file supp_29_1_117__index.html]

Dragon TIS Spotter: an Arabidopsis-derived predictor of translation initiation sites in plants — Supplementary Data 

# Dragon TIS Spotter: an Arabidopsis-derived predictor of translation initiation sites in plants

## Supplementary Data

files

**Files in this Data Supplement:**

- Supplementary Data - pdf file
- Supplementary Data - pdf file
- Supplementary Data - pdf file
- Supplementary Data - jpg file
- Supplementary Data - jpg file
