# Supplementary material for: Dragon TIS Spotter: an Arabidopsis-derived predictor of translation initiation sites in plants
Source: Bioinformatics. 2012 Oct 30;29(1):117–8. doi: 10.1093/bioinformatics/bts638 (PMC3530916; doi:10.1093/bioinformatics/bts638)
Supplement: Supplementary Data [file supp_bts638_Supplementary_Material_1.pdf]

Application Note

DRAGON TIS SPOTTER: AN ARABIDOPSIS-DERIVED PREDICTOR OF TRANSLATION INITIATION SITES WITHIN GENOMIC DNA SEQUENCE IN PLANTS

Arturo M. Mora[1,*], Haitham Ashoor[1,*], Boris R. Jankovic[1,*], Allan Kamau[1], Karim Awara[1], Rajesh Chowdhary[2], John A.C. Archer[1], Vladimir B. Bajic[1, §]

[1]King Abdullah University of Science and Technology (KAUST), Computational Bioscience Research Center, Thuwal 23955-6900, Saudi Arabia.

[2] Biomedical Informatics Research Center, MCRF, Marshfield Clinic, 1000 North Oak Avenue, Marshfield, WI 54449, USA

*These authors contributed equally to this work

[§]Corresponding author: vladimir.bajic@kaust.edu.sa

SUPPLEMENTARY MATERIAL 1: DESCRIPTION OF MAJOR SELECTED FEATURES

The features we considered belong to several broad categories. These include various frequency-based properties of the TIS surrounding sequences or, more generally successive k-mers of nucleotides, scores generated by position weight matrices (PWMs), etc. Another class of features was derived from statistics related to codon biases. In addition, information gain was also considered as a feature of the surrounding sequences. Finally, a number of motifs in ATG-surrounding regions were identified by Dragon Motif Finder (Huang et al. 2005, Marchand et al., 2011), a tool that identifies families of similar short motifs present in the DNA that can be used to enhance the classification process. Out of several hundred features considered, a total of 47 were selected that resulted in the best model performance that was measured in terms of accuracy, defined as Acc=(TP+TN)/(TP+TN+FP+FN), where TP, TN, FP and FN are the numbers of true positive predictions, true negative predictions, false positive predictions and false negative predictions, respectively. The complete list is presented in Supplementary Material 2 accessible at http://cbrc.kaust.edu.sa/dts/code/FeatureList.tar.gz. Below we describe in some detail several of the more prominent among them.

K-mer Frequencies: these represent frequencies of nucleotides and dinucleotides in sequences, separately reported for upstream and downstream regions from the ATG signal. This process generates 40 features.

We introduce two features referred to as Pscore and Nscore, that are calculated as follows: using frequencies of 16 dinucleotide combinations of A, C, T and G we create two PWMs (one from positive and one from negative samples), thus generating two features per sample. The values for Pscore and Nscore are calculated as follows:

Let $S(c_j)$ be a sequence of length L, and P (p_{ij}) is a PWM of L-1 columns and 16 rows (r_1, r_2, r_3,…, r_{16}). The Nscore (from negative data) and Pscore (from positive data) is calculated as follows:

$$[P/N]Score = \sum_{i=1}^{16}\sum_{j=1}^{L-1}\log_2\left(\frac{p_{ij}\otimes c_j c_{j+1}}{Pb_i}\right)$$

$$p_{ij}\otimes c_j c_{j+1} = \begin{cases} p_{ij}, & c_j c_{j+1} = r_i \\ 1, & c_j c_{j+1} \neq r_i \end{cases}$$

where Pb_i stands for the background probability from a uniform distribution.

Kozak's Feature: we utilized the Kozak's consensus sequence that was proposed in (Kozak, 1987). This feature is built on the observation that around the TIS it is highly probable to find an A or G in position -3 and a G in position +4. We check if our sample sequence conforms to GCC[A/G]CCatgG regular expression and if so we assign this binary feature a value of one. Otherwise, we assign to it the value of zero. This feature, whilst useful, is not sufficiently discriminative on its own for the accurate prediction of TIS (Ma et al., 2006). The analysis on our own data, summarized in Supplementary Figure 1 and Supplementary Figure 2, confirms this finding. In addition to this binary Kozak-derived feature, we also use the score based on the Kozak's consensus sequence that represents the number of positions that coincide with the Kozak's consensus sequence. For example, if the example sequence were GCCTCAatgG, the consensus score would be 5, as those nucleotides that are underlined are not expected at their position according to the Kozak's rule.

ATG frequencies: we consider the following three features derived from ATG frequencies:
(1) Number of ATG "in-frame" triplets of non-overlapping nucleotides upstream (the in-frame triplets can appear only downstream of the ATG signal, but we artificially designated the upstream equivalents as being "in-frame"): the number of occurrences of ATG triplets at positions that are "in-frame" aligned. With reference to ATG starting position as 1, these would be occurrences of ATG triplets at positions -3, -6, etc. towards 5' end of the sequence counting the A in ATG as position zero.
(2) Number of ATG "out-of-frame" nucleotide triplets upstream (see explanations above for the "in-frame" upstream designation): the number of occurrences of ATG triplets at positions other than those defined above as upstream "in-frame".
(3) Total number of ATG nucleotide triplets in the sample sequence.

G-quadruplets frequencies: inspired by the work done by Leong and Li (Li and Leong, 2005) in which they state that there is an elevation of G in the downstream section, our analysis shows that it is highly probable to have G-quadruplets in downstream for positive TIS sequences. This process generates two features with the number of downstream G-quadruplets in-frame and out-frame aligned to the ATG segment.

Putative coding sequence: this is a binary feature that indicates whether the sequence contains an in frame stop codon (TAG, TAA or TGA). The idea behind this feature is that since most proteins are longer than 50 amino acids it is less likely that a positive sample would contain a stop codon in frame downstream of ATG. By the same reasoning, there is higher probability to find one of the stop codons in negative samples.

Information Gain: graphs representing frequencies of nucleotides at specific locations in our 300 nucleotide-long positive and negative sequences are presented in Supplementary Fig. 1 and Supplementary Fig. 2, respectively. The shape of these graphs suggests a generally higher level of entropy for negative sequences compared to the positive ones. In order to utilize this observation, for a given position P in the training sequence we calculate the entropy for a nucleotide N as:

$E(P,N) = - p/(p+n) \log2(p/(p+n)) - n/(n+p)\log2(n/(n+p))$

where p represents the number of occurrences of nucleotide N at position P in positive sequences and n represents the number occurrences of the same nucleotide at the position P in negatives sequences. We also introduce another entropy measure at position P that adjusts for the proportion of positive and negative samples in the training set in the following way:

$E(P) = - p/(p+n) \log2(p/(p+n)) - n/(n+p)\log2(n/(n+p))$,

where p and n this time mean the number of positive and negative samples in the training set, respectively. In our case, as we use positive and negative datasets of equal size, the value of E(P) is typically 1.
We use information gain for a position P is defined in (Russel and Norvig, 2008):

$Gain(P) = E(P) – E(A,P) – E(C,P) – E(G,P) – E(T,P)$.

The sum of information gains for the entire sequence (maximum information gain) is then utilized as an input (feature) into the ANN.

C and G versus A and T frequencies: this feature arises from an observation that the frequency of nucleotides C and G is greater than that of A and T in the upstream part of the positive samples. In (Ma et al., 2006) a similar feature is proposed. In our study we compare C and G frequencies to A and T frequencies in the twenty positions that have the highest information gain resulting in four features, namely frequencies of A, C, G and T. Specifically excluded from this feature are those positions with the highest information gain as they are already contained in the Kozak feature.

In-frame nucleotides score: loosely inspired by the position-specific k-gram approach that was used in (Liu and Wong, 2003). We first determine "in frame" triplets (as described previously) and within each of these triplets we note the numbers of As, Cs, Ts and Gs that are located in each of the three positions within triplets. We then sum up for each nucleotide the cumulative number of it occurrences in positions 1, 2 and 3 within triplets. These sums are then taken as feature values. Since there are four nucleotides and three positions, there are 12 feature values arising in this way. Since we count separately those in frame downstream and those "in frame" upstream (as described above), the total number of features obtained in this way are 24. As an example, if we consider the sequence ATGattgcc we would identify 2 in frame (as it is downstream) triplets (att and gcc) and then count the number of occurrences of each of the nucleotides: for position 1, we have one 'a', one 'g' and zero 'c' and 't'. For the second position, we have one 't', one 'c' and zero 'a' and 'g'. For the third position, we have one 'c', one 't' and zero "a" and 'g'. Therefore, the feature values would be, referenced to sequence acgt the following: 1,0,1,0,0,1,0,1,0,1,0,1.

Motifs present: using Dragon Motif Finder tool (Huang et al. 2005, Marchand et al., 2011) (http://apps.sanbi.ac.za/dmb), we identified a number of motif families of various lengths that are present in the upstream, central, and downstream regions surrounding the candidate TIS motif in positive samples using negative data as a background, as described in (Huang et al. 2005). These regions are defined as follows: the upstream region contains 150 nucleotides between the 5' end of the sequence and ATG triplet, the downstream region contains 147 nucleotides between ATG triplet and the 3' end of the sequence and the central region contains nucleotides 50 upstream before ATG motif and 50 downstream after ATG motif. Presence of sequences of these motif families is then used as a feature for *Arabidopsis thaliana.* model. We considered 20 motifs for upstream, central and downstream regions independently, generating a total of 60 features.

In this work, each of our features is independently normalized on the training data to values in the range [-1, 1] in order to improve the performance of ANN training (Han and Kamber, 2006). As a result our feature selection process selected 47 features for A.t. model.

SUPPLEMENTARY MATERIAL 1: FEATURE SELECTION PROCESS

The feature selection process is aimed at determining an optimal set of features out of all candidate model features in order to derive models with smaller number of parameters that should be more robust and less prone to overtraining. The staring point in our feature selection process is the definition of a predetermined set of features. This set is fixed in the sense that it always forms a part of the selected features set all iterations. The features in this set were determined on the basis of the highest gain ratio reported by gain ratio attribute evaluator in Weka tool (Hall et al., 2009) and those selected are: Pscore, Nscore, Kozak's feature, ATG frequency, putative coding sequence, frequency of "t" nucleotide in downstream in frame codons in position 3, "ag" and "cc" dinucleotide frequency in the upstream segment and "dw" dinucleotide frequency in downstream segment (altogether 10 feature).
The core step in our feature selection process is the application of genetic algorithm (GA) in search of an optimal features combination. Briefly, the process stipulates that all candidate features are numbered and assigned a value of 0 (not selected as a member of a feature set) or 1 (selected). In this way we form a "chromosome" in the GA terms. We use a single point crossover together with mutation where each bit in a chromosome is subjected to 15 percent chance of having its value altered. Finally, we define evaluation function as the accuracy of model based on a 3-fold cross-validation on the training data.
The process works as follows. Within each GA iteration, the training set (12,802 positive and 12,802 negative samples) is divided into the validation set (1,920 positive and 1,920 negative samples) and the remaining 10,882 positive and 10,882 negative samples are used to form the three folds of the 3-fold cross validation. An ANN model (using the features indicated by the content of the chromosome of the current iteration) is trained using positive and negative data in two of the folds. Early stopping with validation, utilising the validation set, is deployed to determine the stopping point of training as well as to prevent model over-training. The model is then tested on the data in the remaining fold and its accuracy recorded. The average accuracy of the 3-fold cross validation is then used as the fitness function of the GA. This process is repeated until the predefined number of GA iterations (we used 150) is reached.

SUPPLEMENTARY MATERIAL 1 REFERENCES

Hall M, Frank E, Holmes G, Pfahringer B, Reutemann P, Witten IH (2009); The WEKA Data Mining Software: An Update; SIGKDD Explorations, Volume 11, Issue 1.
Han J and Kamber M. (2006) Data Mining: Concepts and Techniques, Second Edition. Morgan Kufmann.
Huang, L Yang, Chowdhary R, Kassim A, Bajic VB. (2005) An algorithm for ab initio DNA motif detection. In Information Processing and Living Systems, World Scientific, 611-614.
Kozak M. (1989) Context effects and inefficient initiation at non-AUG codons in eucaryotic cell-free translation systems. Mol. Cell. Biol. 9:5073–5080.
Liu H and Wong L. (2003) Data Mining Tools for Biological Sequences, J. Bioinformatics and Computational Biology. 1(1):139-167.
Ma C, Zhou D, Zhou Y. (2006) Feature Mining Integration for Improving the Prediction Accuracy of Translation Initiation Sites in Eukaryotic mRNAs. gccw, 349-356, Fifth International Conference on Grid and Cooperative Computing Workshops.
Marchand B, Bajic VB, Kaushik D. (2011) Highly Scalable Ab Initio Genomic Motif Identification, SuperComputing 2011, International Conference for High Performance Computing, Networking, Storage and Analysis, Seattle 15-22 November 2012
Russel S and Norvig P. (2003) Artificial Intelligence: A Modern Approach, Second Edition, Prentice-Hall, pp. 659-660.
Stormo GA. (2000) DNA binding sites: representation and discovery. Bioinformatics, vol. 1, pp. 16-23.
