# Supplementary material for: Dragon TIS Spotter: an Arabidopsis-derived predictor of translation initiation sites in plants
Source: Bioinformatics. 2012 Oct 30;29(1):117–8. doi: 10.1093/bioinformatics/bts638 (PMC3530916; doi:10.1093/bioinformatics/bts638)
Supplement: Supplementary Data [file supp_bts638_Supplementary_Material_2.pdf]

Application Note

DRAGON TIS SPOTTER: AN ARABIDOPSIS-DERIVED PREDICTOR OF TRANSLATION INITIATION SITES WITHIN GENOMIC DNA SEQUENCE IN PLANTS

Arturo M. Mora[1,*], Haitham Ashoor[1,*], Boris R. Jankovic[1,*], Allan Kamau[1], Karim Awara[1], Rajesh Chowdhary[2], John A.C. Archer[1], Vladimir B. Bajic[1, §]

[1]King Abdullah University of Science and Technology (KAUST), Computational Bioscience Research Center, Thuwal 23955-6900, Saudi Arabia.

[2] Biomedical Informatics Research Center, MCRF, Marshfield Clinic, 1000 North Oak Avenue, Marshfield, WI 54449, USA

*These authors contributed equally to this work

[§]Corresponding author: vladimir.bajic@kaust.edu.sa

SUPPLEMENTARY MATERIAL 2: LIST OF FEATURES USED IN DRAGON TIS SPOTTER FOR ARABIDOPSIS THALIANA

The following table provides a list of all features used in Dragon TIS Spotter for plants. The names given to the features correspond to the names of features as used in the programs implementing the tool. Features are derived from Arabidopsis data sets.

pscore	Positive score value calculated based on PWM
nscore	Negative score value calculated based on PWM
kozak	Binary value indicating the conservation of the consensus GCC[A/G]CCatgG
G_ratio	Number of G nucleotides in the 20 upstream posititions with the highest information gain
UpstreamATGin	In-frame ATG condon frequency in the upstream segment
UpstreamATGout	Out-frame ATG condon frequency in the upstream segment
Num_ATG	ATG codon frequency in both upstream and downstream segments
putative_sequence	"Binary value that indicates whether the sequence contains an in-frame stop codon (TAG TAA or TGA)"
Info_gain	Information gain value calculated based on the entropy of the nucleotides in the sequence
u_kth1_a	Frequency of nucleotide A in position 1 in the in-frame codons for the upstream segment
u_kth2_a	Frequency of nucleotide A in position 2 in the in-frame codons for the upstream segment
u_kth3_a	Frequency of nucleotide A in position 3 in the in-frame codons for the upstream segment
u_kth2_c	Frequency of nucleotide C in position 2 in the in-frame codons for the upstream segment
u_kth3_c	Frequency of nucleotide C in position 3 in the in-frame codons for the upstream segment
u_kth2_g	Frequency of nucleotide G in position 2 in the in-frame codons for the upstream segment
u_kth2_t	Frequency of nucleotide T in position 2 in the in-frame codons for the upstream segment
d_kth1_a	Frequency of nucleotide A in position 1 in the in-frame codons for the downstream segment
d_kth2_g	Frequency of nucleotide G in position 2 in the in-frame codons for the downstream segment
d_kth2_t	Frequency of nucleotide T in position 2 in the in-frame codons for the downstream segment
upstream_a	1-gram feature: frequency of A nucleotide in the upstream segment
upstream_c	1-gram feature: frequency of C nucleotide in the upstream segment
upstream_t	1-gram feature: frequency of T nucleotide in the upstream segment
downstream_a	1-gram feature: frequency of A nucleotide in the downstream segment
downstream_c	1-gram feature: frequency of C nucleotide in the downstream segment
downstream_g	1-gram feature: frequency of G nucleotide in the downstream segment
downstream_t	1-gram feature: frequency of T nucleotide in the downstream segment
upstream_2mer_aa	2-fram feature: frequency of AA dinucleotides in the upstream segment
upstream_2mer_ac	2-fram feature: frequency of AC dinucleotides in the upstream segment
upstream_2mer_at	2-fram feature: frequency of AT dinucleotides in the upstream segment
upstream_2mer_ca	2-fram feature: frequency of CA dinucleotides in the upstream segment
upstream_2mer_cg	2-fram feature: frequency of CG dinucleotides in the upstream segment
upstream_2mer_gc	2-fram feature: frequency of GC dinucleotides in the upstream segment
upstream_2mer_gt	2-fram feature: frequency of GT dinucleotides in the upstream segment
upstream_2mer_tt	2-fram feature: frequency of TT dinucleotides in the upstream segment
downstream_2mer_ag	2-fram feature: frequency of AG dinucleotides in the downstream segment

© Oxford University Press 2005

downstream_2mer_at	2-fram feature: frequency of AT dinucleotides in the downstream segment
downstream_2mer_ca	2-fram feature: frequency of CA dinucleotides in the downstream segment
downstream_2mer_cc	2-fram feature: frequency of CC dinucleotides in the downstream segment
downstream_2mer_cg	2-fram feature: frequency of CG dinucleotides in the downstream segment
downstream_2mer_ct	2-fram feature: frequency of CT dinucleotides in the downstream segment
downstream_2mer_gc	2-fram feature: frequency of GC dinucleotides in the downstream segment
downstream_2mer_gg	2-fram feature: frequency of GG dinucleotides in the downstream segment
downstream_2mer_gt	2-fram feature: frequency of GT dinucleotides in the downstream segment
downstream_2mer_tg	2-fram feature: frequency of TG dinucleotides in the downstream segment
upstream DMF	Accumulative frequency of the 20 most common motifs in the upstream segment
downstream DMF	Accumulative frequency of the 20 most common motifs in the downstream segment
middle DMF	Accumulative frequency of the 20 most common motifs in the segment around the candidate TIS (50 nucleotides downstream and 50 upstream)
