# Supplementary material for: Dragon TIS Spotter: an Arabidopsis-derived predictor of translation initiation sites in plants
Source: Bioinformatics. 2012 Oct 30;29(1):117–8. doi: 10.1093/bioinformatics/bts638 (PMC3530916; doi:10.1093/bioinformatics/bts638)
Supplement: Supplementary Data [file supp_bts638_Supplementary_Material_3.pdf]

*Application Note*

# DRAGON TIS SPOTTER: AN ARABIDOPSIS-DERIVED PREDICTOR OF TRANSLATION INITIATION SITES WITHIN GENOMIC DNA SEQUENCE IN PLANTS

Arturo M. Mora[1,*], Haitham Ashoor[1,*], Boris R. Jankovic[1,*], Allan Kamau[1], Karim Awara[1], Rajesh Chowdhary[2], John A.C. Archer[1], Vladimir B. Bajic[1, §]

[1]King Abdullah University of Science and Technology (KAUST), Computational Bioscience Research Center, Thuwal 23955-6900, Saudi Arabia.

[2] Biomedical Informatics Research Center, MCRF, Marshfield Clinic, 1000 North Oak Avenue, Marshfield, WI 54449, USA

*These authors contributed equally to this work

[§]Corresponding author: vladimir.bajic@kaust.edu.sa

## SUPPLEMENTARY MATERIAL 3: TESTING OF DRAGON TIS SPOTTER ON ARABIDOPSIS AND OTHER PLANT GENOMES

In addition to reporting DTS prediction results TISs in Arabidopsis on the test set, as described in the text of the main manuscript and the Supplementary Material 1, we also tested the predictions of the tool on the entire Arabidopsis genome, with exception of those TIS sites in the genome that were used for ANN weights training, feature generation and early stopping with validation. For this test we achieved sensitivity of 90.75% and specificity of 92.25%. This test was performed in order to avoid any bias in selection of the test dataset.

Dragon TIS Spotter, in the exactly the same form as derived from Arabidopsis thaliana genomic datasets was tested on genomic sequences of two other plant genomes, *Vitis vinifera* and *Populus trichocarpa*. These genomes and genome annotations are downloaded and assembled from Solanaceae Genomics Resource at Michigan State University (http://solanaceae.plantbiology.msu.edu) in period 11-12 Jun 2012. Specifically, sequences and annotations for grape were downloaded from http://solanaceae.plantbiology.msu.edu/cgi-bin/gbrowse/grape and for poplar from http://solanaceae.plantbiology.msu.edu/cgi-bin/gbrowse/poplar. Because we consider only those sites that start with ATG, we eliminated from our test data any non-conforming annotated TIS sites for both plants.

In order to avoid any bias in testing, TIS predictions were performed on entire chromosomes 1 and 2 in case of grape, and on the entire chromosome 1 in the case of poplar. Our tool's performance on grape and poplar data are sensitivity of 66.8% and 81.6% and specificity of 94.45% and 94.4%, respectively. Details are given in the table below:

### Supplementary table 1: DTS prediction performance on grape and poplar

| Chromosome | Total #ATG | Total predicted TIS | True TIS | Predicted True TIS | Sensitivity | Specificity |
|---|---|---|---|---|---|---|
| Grape #1 | 627733 | 35632 | 762 | 504 | 66.1% | 94.4% |
| Grape #2 | 681884 | 37988 | 684 | 462 | 67.5% | 94.5% |
| Poplar #1 | 1266018 | 72484 | 1573 | 1284 | 81.6% | 94.4% |

These results indicate that DTS can be successfully used as a tool for genome annotation in other, non-model plant genomes.

© Oxford University Press 2005
