# Supplementary figures and images for: Dragon TIS Spotter: an Arabidopsis-derived predictor of translation initiation sites in plants
Source: Bioinformatics. 2012 Oct 30;29(1):117–8. doi: 10.1093/bioinformatics/bts638 (PMC3530916; doi:10.1093/bioinformatics/bts638)

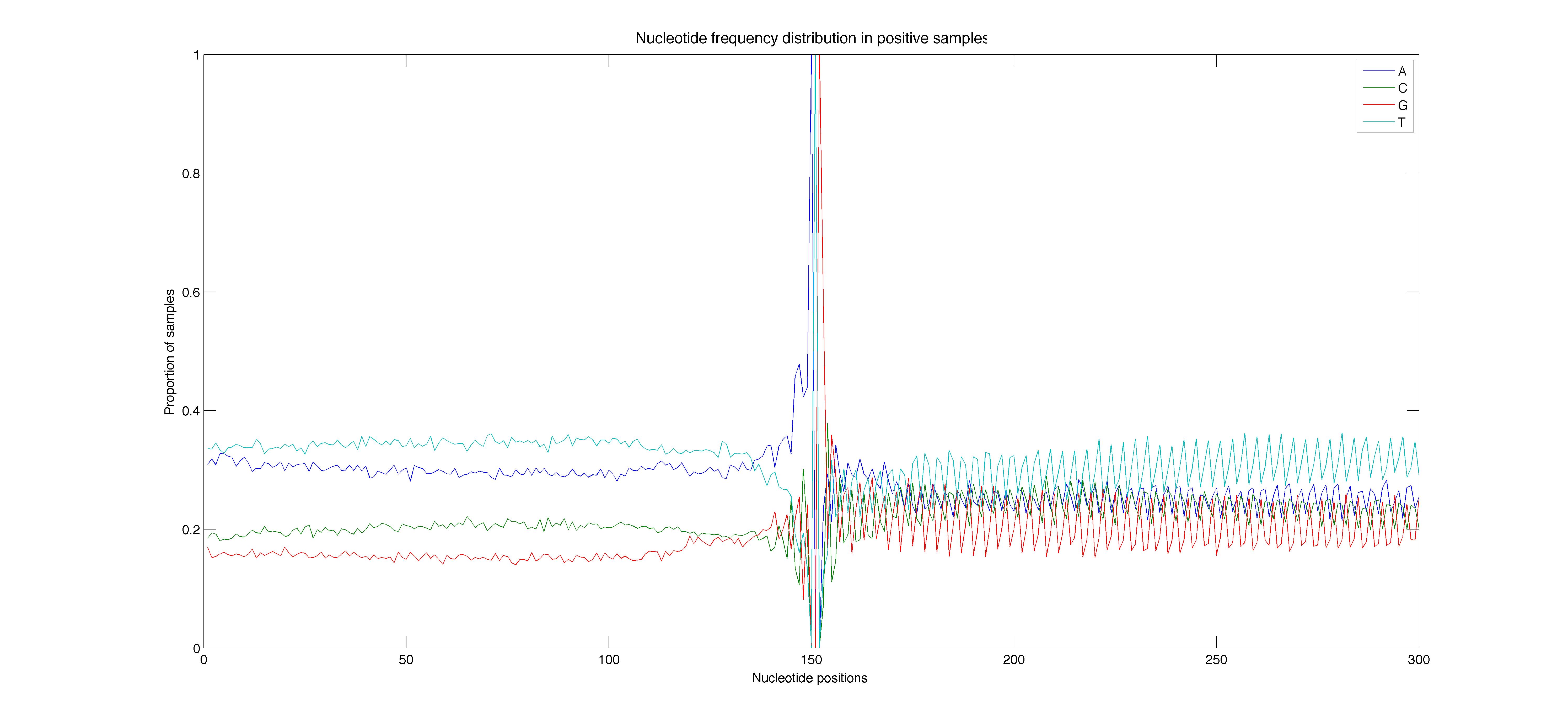

Supplement: Supplementary Data [file supp_bts638_Supplementary_1_Figure_1.jpg]

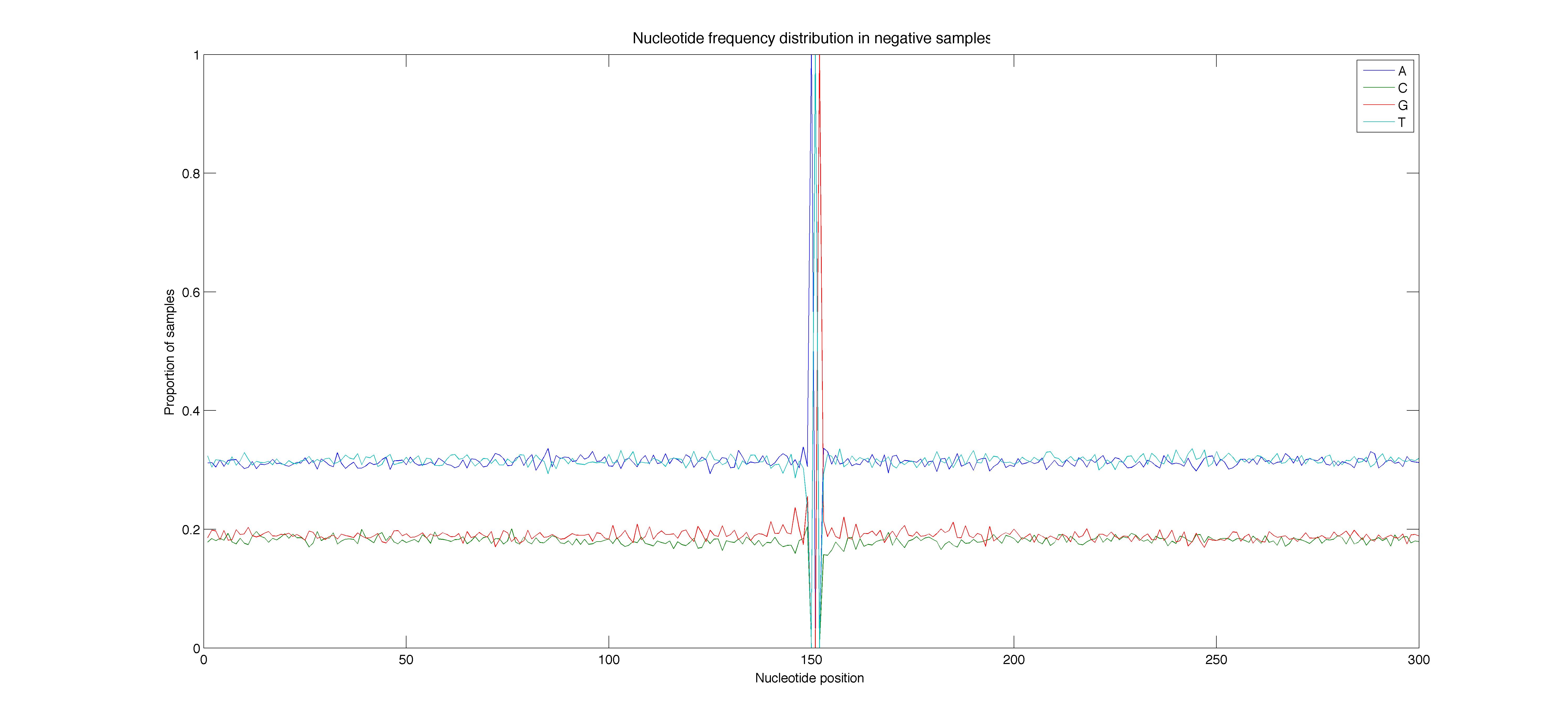

Supplement: Supplementary Data [file supp_bts638_Supplementary_1_Figure_2.jpg]
